# Engineering meiotic recombination pathways in rice

**DOI:** 10.1111/pbi.13189

**Published:** 2019-07-02

**Authors:** Ian Fayos, Delphine Mieulet, Julie Petit, Anne Cécile Meunier, Christophe Périn, Alain Nicolas, Emmanuel Guiderdoni

**Affiliations:** ^1^ Cirad UMR AGAP Montpellier France; ^2^ Université de Montpellier Cirad-Inra-Montpellier SupAgro Montpellier France; ^3^ Institut Curie, CNRS UMR 3244 University PSL Paris France; ^4^ Meiogenix Paris France

**Keywords:** apomixis, crossovers, meiosis, recombination, rice

## Abstract

In the last 15 years, outstanding progress has been made in understanding the function of meiotic genes in the model dicot and monocot plants *Arabidopsis* and rice (*Oryza sativa* L.), respectively*.* This knowledge allowed to modulate meiotic recombination in *Arabidopsis* and, more recently, in rice. For instance, the overall frequency of crossovers (COs) has been stimulated 2.3‐ and 3.2‐fold through the inactivation of the rice *FANCM* and *RECQ4* DNA helicases, respectively, two genes involved in the repair of DNA double‐strand breaks (DSBs) as noncrossovers (NCOs) of the Class II crossover pathway. Differently, the programmed induction of DSBs and COs at desired sites is currently explored by guiding the *SPO11‐1* topoisomerase‐like transesterase, initiating meiotic recombination in all eukaryotes, to specific target regions of the rice genome. Furthermore, the inactivation of 3 meiosis‐specific genes, namely *PAIR1*, *OsREC8* and *OsOSD1*, in the *Mitosis instead of Meiosis* (*MiMe*) mutant turned rice meiosis into mitosis, thereby abolishing recombination and achieving the first component of apomixis, apomeiosis. The successful translation of *Arabidopsis* results into a crop further allowed the implementation of two breakthrough strategies that triggered parthenogenesis from the *MiMe* unreduced clonal egg cell and completed the second component of diplosporous apomixis. Here, we review the most recent advances in and future prospects of the manipulation of meiotic recombination in rice and potentially other major crops, all essential for global food security.

## Introduction

Rice is a staple food for more than half of mankind. It is estimated that current rice production should be raised by 50% to meet the demand for the 2050 world population, which will mainly occur in rice‐eating countries (Alexandratos and Bruinsma, 2012). Genetic improvement together with agricultural practices greatly contributed to the overall yield gain accomplished from 1960 to 2010, which doubled the average yield from 2 to 4 t/ha and saved an estimated 250 million ha of land from cultivation (Williams, 2017). However, since the early 2000s, rice and other cereal crops yields have reached a plateau (Grassini *et al.*, 2013). These plateaus are partly due to enhanced climate instability and insufficient crop rotation (Bennett *et al.*, 2012). There is therefore an urgent need to develop new varieties with enhanced yield potential, lower water, chemical fertilizer and protection demands as well as genetic tolerance to abiotic constraints and extreme climatic events and ultimately a reduced contribution to greenhouse gas emission (Weller *et al.*, 2016).

In the coming decade, genomics and genome editing tools will likely assist in the development of new cultivars through precision engineering technologies and improved breeding schemes (Li *et al.*, 2018a). Advances will be facilitated by knowledge of the nucleotide variation of accessions in the two cultivated species (*Oryza sativa *L. and *Oryza glaberrima* Steud.) and their wild relatives (Stein *et al.*, 2018; Wang *et al.*, 2014, 2018; Zhao *et al.*, 2018) as well as of the functional characterization of their genes (Li *et al.*, 2018b). The starting raw material for breeders is allelic variation that is naturally reshuffled upon meiotic recombination and transmitted by the gametes. This creates novel allelic combinations harnessed by breeders to create improved phenotypes. However, meiotic recombination between the homologous chromosomes is hampered by the restricted number of COs per chromosome (typically 1–3 per chromosome pair) and globally per meiotic cell (Mercier *et al.*, 2015). Additionally, recombination frequency may vary up to 100‐fold across regions in large plant genomes (Mézard *et al.*, 2015), limiting access to genes of interest residing in ‘cold’ recombination regions. Thus, making meiosis amenable to manipulation for enhancing and/or targeting recombination is highly desirable.

The process of meiosis has a dual role: to generate the genetic diversity transmitted by the gametes but also to ensure proper segregation of the chromosomes into the gametes. Defects in recombination and meiosis are a major source of sterility. As in all eukaryotes, the reductional and equational meiotic divisions reduce the number of chromosomes transmitted by the gametes by half. Then, fertilization allows a return to the species chromosome number. However, some plant species exhibit clonal asexual reproduction via seeds, a mode of reproduction called apomixis (Koltunow and Grossniklaus, 2003). A major mode of apomixis in grasses is gametophytic, diplosporous apomixis, which bypasses meiosis through the formation of an embryo from the unreduced egg cell, resulting in clonal progeny identical to the maternal parent. Transferring this mode of reproduction to crops will have the considerable advantage of propagating hybrid vigour across generations via seeds. The possibility of creating apomictic hybrids in rice, a self‐propagated crop, will allow heterosis in the crops of subsistence farmers. To achieve this, long‐term goal requires to abolish recombination in F1 hybrids, that is turning meiosis into mitosis, and to trigger parthenogenetic development from the unreduced egg cell.

The isolation of numerous meiosis mutants and characterization of their functions in model species such as budding yeast (Keeney *et al.*, 2014), *Caenorhabditis* (Hillers *et al.*, 2017; Yu *et al.*, 2016) and the plant *Arabidopsis* (Lambing *et al.*, 2017; Mercier *et al.*, 2015; Wang and Copenhaver, 2018) have been fruitful. Similarly, over the last 15 years, functional approaches have been intensively pursued in rice. In a 2014 survey celebrating 10 years of accomplishments, Luo *et al.*, (2014) identified 28 functionally characterized meiotic genes in rice. These genes are involved in the entry into meiosis, sister chromatid cohesion, protection of centromeric cohesion, formation and processing of the recombination initiating DNA double‐strand breaks (DSBs), strand invasion/exchange, synaptonemal complex formation and resolution of the recombination intermediates leading to NCO and CO recombinant molecules. Since, the function of more than 20 other rice meiotic genes has been uncovered, making rice a major contributor to the nearly 90 meiotic genes characterized in plants to date (Wang and Copenhaver, 2018). As a complement to Luo’s and collaborators' review, we will focus hereafter on recent achievements in the engineering of meiotic recombination pathways in rice. Engineering aimed at either enhancing or targeting recombination or, conversely, at abolishing recombination to produce unreduced egg cells that can serve for triggering parthenogenesis and thus achieving apomixis.

## Meiosis and recombination

**Figure 1:**
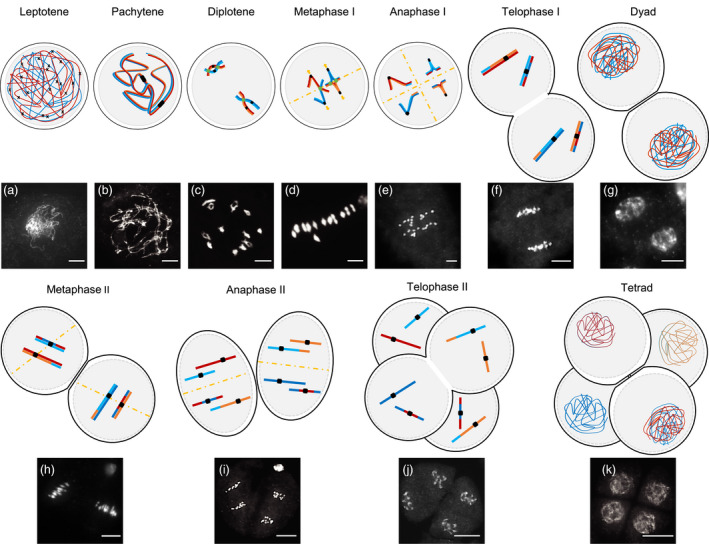
4′,6‐diamidino‐2‐phenylindole (DAPI)‐stained chromosomes and schematic representation of pollen mother cells during male meiosis progression in wild‐type rice. Meiosis I and II encompass 4 phases: prophase, metaphase, anaphase and telophase. Prophase I is extended and subdivided into 5 stages. (a) In leptotene, duplicated sister chromatids start condensation, forming thin thread‐like structures, while the induction of double‐strand breaks (DSBs) (shown as black crosses) initiates recombination. This process is followed by the installation of the synaptonemal complex (SC) and initiation of synapsis in zygotene. (b) Fully synapsed homologous chromosomes appearing as condensed, thick, thread‐like structures are observed in pachytene: meiotic DSBs have been repaired. (c) In diplotene, SC disassembly takes place, and homologs partially separate, except in chiasmata (shown as green circles), where COs have occurred. Highly condensed chromosomes paired bivalently can be observed at diakinesis: homologs are still linked by chiasmata and sister chromatid cohesion. A coordinated change in sister chromatid cohesion and kinetochore orientation will allow segregation of homologous chromosomes and then of sister chromatids during meiosis I and meiosis II, respectively. Prophase of meiosis is followed by metaphase I (d), with the alignment of bivalents along the equatorial plate (showed as yellow dashed line). The next phases are anaphase I (e), where homologous chromosomes separate by migrating to opposite poles, and telophase I. (f) Chromosomes condense at the dyad stage (g), which is immediately followed by the mitosis‐like meiosis II division metaphase II (h) and sister chromatid separation in anaphase II (i) and telophase II (j). Meiosis is completed with the formation of tetrads (k) containing a halved, recombined chromosome complement. Bar = 10 micrometres. The fate of two pairs of homologous chromosomes is represented for simplification. The centromeres that contain the kinetochore connecting chromosomes to the spindle, allowing segregation, appear as dark squares.

Meiosis is a specialized cell differentiation that leads to the formation of gametes containing half of the species chromosomal complement. This ploidy reduction is ensured by a single round of DNA replication followed by two rounds of chromosome segregation: first, the reductional division (MI) that segregates the homologous chromosomes and second (MII) the equational segregation that separate the sister chromatids (Figure 1). During anaphase I, the sister chromatid cohesion is released along the chromosome arms and the kinetochores are oriented towards the same pole, allowing the accurate migration and segregation of the homologous chromosomes. Then, during anaphase II, the pericentromeric cohesion is released and the kinetochores of the sister chromatids are oriented towards opposite poles, allowing the separation of the sister chromatids and the formation of four cells (tetrads).They will finally differentiate to generate the gametes. Meiotic recombination that occurs during the prophase of MI after DNA replication generates two classes of recombination products. The recombinant molecules issued from a crossover (CO) event result from a reciprocal exchange between two non‐sister chromatids carried by the homologous chromosomes. Its cytological manifestation is the chiasmata. In contrast, the recombinant molecules issued from a noncrossover event (NCO) locally acquire a small stretch of DNA from the homologous chromosome, without exchange of the flanking markers. When the four products of a single meiosis can be recovered in tetrads, this unidirectional transfer of information called a ‘gene conversion’. It is associated or not to an adjacent crossover. Thus, recombination between the polymorphic paternal and maternal chromosomes in hybrids leads to novel combination of alleles transmitted by the gametes and potentially encoding new traits in the progenies. The other essential role of the crossover events is to ensure a physical linkage between the homologous chromosomes, allowing their proper segregation at the reductional division (Mercier *et al.*, 2015). In the absence of COs, the homologs segregate randomly, a source of infertility. Thus, the formation of balanced and viable gametes requires an ‘obligate CO’ per chromosome pair, although few additional COs may occur leading to 1–3 COs per chromosome pair per meiosis in most eukaryotes (Fernandes et al., 2018a).

**Figure 2:**
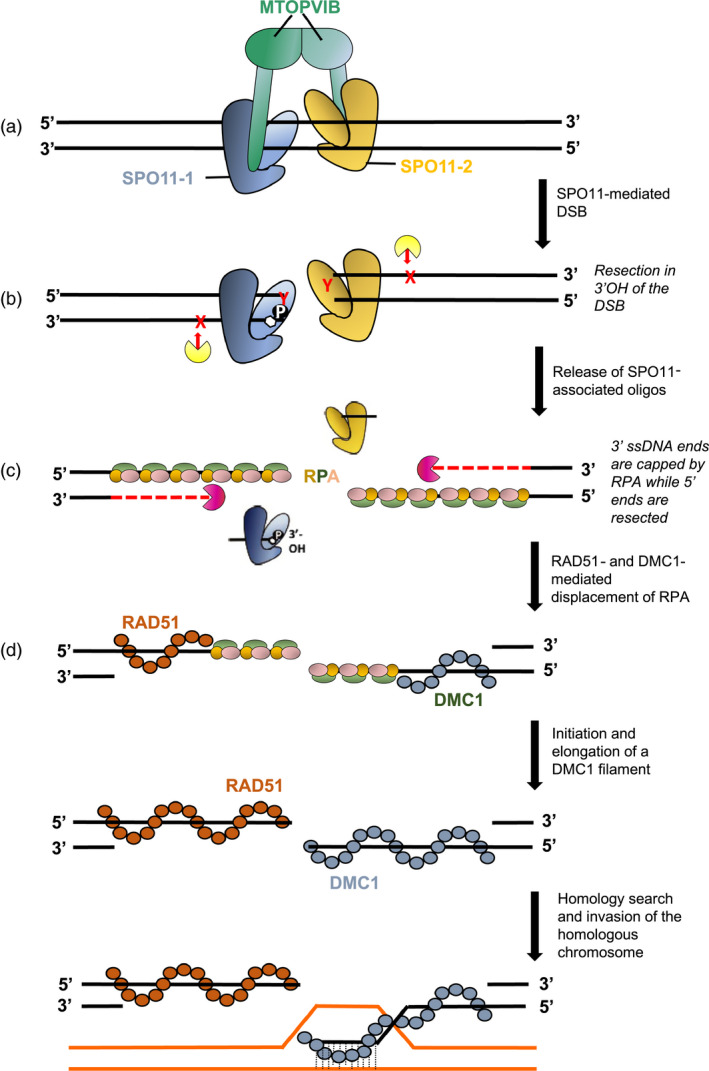
SPO11 initiates recombination by inducing chromosomal double‐strand breaks (DSBs). (a) The current model in flowering plants combines the transesterases SPO11‐1 and SPO11‐2 and 2 units of the meiosis‐specific topoisomerase M‐TOPOVI‐B into a heterotetramer recruited on the chromatin to induce DSBs (Vrielynck et al., 2016). Accessory proteins are not shown for simplification. (b) While SPO11 proteins remain covalently attached to the 5′ end of the break, 3′OH resection of single‐stranded DNA occurs (endonuclease activity represented in yellow), thereby releasing SPO11‐associated oligonucleotides. In yeast and mammals, this is accomplished by the nucleolytic activity of the MRX‐N complex (Mre11/Rad50/Xrs2(Nbs1)), which is also involved in DSB formation in yeast, together with Com1(Sae2). In *Arabidopsis*, AtMRE11 and AtRAD50 are involved in mitotic and meiotic DNA repair but not in DSB formation, while AtNBS1 is non‐essential to meiosis. AtCOM1 (and its rice ortholog OsCOM1) has a meiotic function similar to AtMRE11 and AtRAD50 and may act together in DNA end processing. (c) The 5′ ends are further resected (exonuclease activity represented in purple) to release 3′ end ssDNA tails on the complementary strand. This function is ensured is by Exo1 and the Sgs1 helicase in yeast. The 3′ends are first bound by a heterotrimeric RPA (RPA 1, 2, 3) protein complex. In plants, a multigenic family encodes each RPA component. (d) The strand is then loaded by the RAD51 and DMC1 recombinases that replace RPAs to form a nucleoprotein filament ready for homology search and heteroduplex formation. Four models have been envisioned to explain how the strand exchange proteins RAD51 and DMC1 cooperate (mixed filaments, co‐filaments of RAD51 and DMC1 patches, temporally separated consecutive loads, asymmetric filaments of RAD51 and DMC1 at each end of the DSB). In plants, cytological evidence of separate loading of RAD51 and DMC1 onto opposite strands at each end of the DSB has been reported (Kurzbauer et al., 2012) and is illustrated here.

The early studies of recombination hot spots in yeast showed that meiotic recombination is initiated by the formation of programmed DNA double‐strand breaks (DSBs), occurring after DNA replication (Nicolas, *et al.*, 1989; Sun *et al.*, 1989). The DSBs are induced by the evolutionarily conserved, topoisomerase‐like transesterase protein SPO11, orthologous of the DNA‐cleaving A subunit of the archae TopoVI DNA topoisomerase (Bergerat *et al.*, 1997; Keeney *et al.*, 1997). *Arabidopsis* has two distinct SPO11 paralog proteins, namely AtSPO11‐1 and AtSPO11‐2, which interact with each other and with the MtopVIB protein, the ortholog of the archae *TopoVIB* subunit (Bergerat *et al.*, 1997; Vrielynck *et al.*, 2016), suggesting the assembly of a heterotetrameric complex (Figure 2). In *Arabidopsis,* the *Atspo11‐1*, *Atspo11‐2* and *MtopVIB* mutants are sterile being defective in meiotic recombination, chromosome pairing and meiosis progression (Grelon *et al.*, 2001; Hartung and Puchta, 2000; Stacey *et al.*, 2006; Vrielynck *et al.*, 2016). Consistently, rice plants deficient in OsSPO11‐1 exhibit defects in homologous chromosome pairing, and their meiocytes do not display CO‐related proteins (Yu *et al.*, 2010). The inactivation of the likely rice orthologue of *AtSPO11‐2*, *OsSPO11‐2*, has not yet been reported but contrasted reports on the occurrence of interaction between OsSPO11‐2 and OsMTOPVIB have been recently published (Fu *et al.*, 2016; Xue *et al.*, 2016). A third SPO11 paralog, AtSPO11‐3, has been identified in *Arabidopsis* and other plants; it plays a role in somatic cells but has no role in meiotic recombination (Hartung *et al.*, 2002). By analogy, OsSPO11‐3, which shares 69% identity with AtSPO11‐3, is expected to be involved in somatic function but the meiotic phenotype of *Osspo11‐3* remains to be determined. Ectopic expression of *OsSPO11‐3* confers salt and osmotic stress tolerance to *Arabidopsis* plants (Jain *et al.*, 2006). A fourth SPO11 paralog, OsSPO11‐4, was identified in rice but not in other plants. It was reported to exhibit DNA cleavage activity *in vitro* (An *et al.*, 2011) as well as in a *Drosophila* assay (Shingu *et al.*, 2012) and to interact with OsMTOPVIB (Fu *et al.*, 2016). *OsSPO11‐4* suppression resulted in defect in male meiosis and reduced fertility (An *et al.*, 2011). The *Osspo11-1, Osspo11-2 and Osspo11-4* mutant phenotypes have been re‐investigated after CRISPR‐Cas9 mutagenesis (Fayos *et al.*, submitted). Not surprisingly, the plants exhibiting detrimental lesions in *OsSPO11‐1* and *OsSPO11‐2* were fully sterile. However, a range of *Osspo11‐4* mutants, targeted in distinct regions of the coding sequence (CDS), were fertile and exhibited a wild‐type meiosis progression, indicating that OsSPO11‐4 is not crucial for meiosis and viable gamete formation.

In yeast, the formation of SPO11‐induced DSBs involves at least nine other proteins (Mre11, Rad50, Xrs2, Ski8, Rec102, Rec104, Rec114, Mei4 and Mer2), some but not all identified yet across kingdoms. In plants, seven proteins have been found required for meiotic DSB formation: AtPRD1, AtPRD2, AtPRD3/PAIR1, AtDFO, OsCRC1, OsSDS and OsP31 COMET (Ji *et al.*, 2016; Miao *et al.*, 2013; De Muyt *et al.*, 2009a; De Muyt *et al.*, 2007; Nonomura *et al.*, 2004; Wu *et al.*, 2015; Zhang *et al.*, 2012). AtPRD1 and AtPRD2 are the likely orthologues of mouse Mei1 and yeast and mouse Mei4, while AtPRD3 (PAIR1 in rice) and OsCRC1 might be plant‐specific. PAIR1, first isolated in rice, has no identified function and no known orthologue outside the plant kingdom (Nonomura *et al.*, 2004). Rice CRC1 (Central Region Component 1) shares 23.1% similarity with yeast Pch2, a member of the conserved AAA + ATPase protein family likely involved in remodelling chromosome structure in the vicinity of DSBs. CRC1 interacts with OsZEP1 in vitro and CRC1‐deficient rice meiocytes are asynaptic with only univalents (Miao *et al.*, 2013). Intriguingly, in Arabidopsis, the AtPch2 mutation does not alter DSB formation while it is required for meiosis (Lambing et al., 2015). Whereas mutation in OsSDS appeared to abolish DSB formation in rice (Wu et al., 2015), this meiosis‐specific cyclin‐like protein is required for DMC1‐mediated DSB repair but not for DSB formation in Arabidopsis (De Muyt et al., 2009a). P31 COMET that interacts with CRC1 is an additional protein essential for DSB formation in rice (Ji *et al.*, 2016).

**Figure 3:**
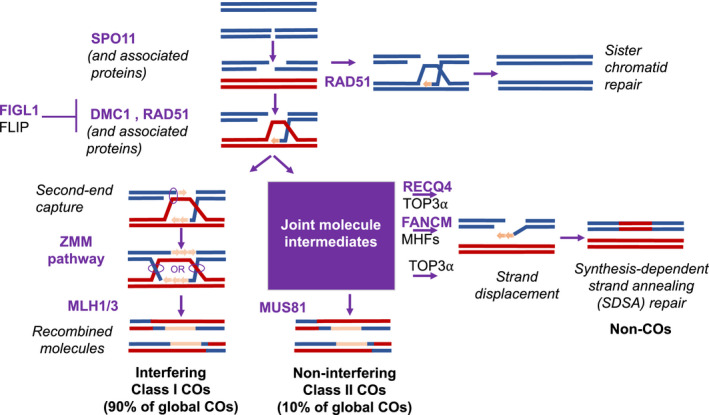
A simplified representation of pathways allowing the formation of meiotic crossovers (COs) and noncrossovers (NCOs). The ZMM‐ and MUS81‐dependent pathways generate COs and NCOs at meiosis following the programmed induction of chromosomal DSBs by the SPO11 complex in the prophase of meiosis. The AAA‐ATPase FIDGETIN‐LIKE 1 (*figl1*) forms a protein complex with its partner FIDGETIN‐LIKE‐1 INTERACTING PROTEIN (FLIP), which counteracts RAD51 and DMC1, the recombinases catalysing the DNA exchange step of homologous recombination (Fernandes, Duhamel, et al., 2018a; Girard et al., 2015). The two DNA helicases Fanconi anaemia of complementation group M (FANCM) (Crismani et al., 2012) and the plant homolog of slow growth repressor 1 (Sgs1)/Bloom syndrome (BLM) protein, RECQ4 (Séguéla‐Arnaud et al., 2015), antagonize COs by processing recombination intermediates in the Class II CO formation pathway (adapted from (Mercier et al., 2015)).

Following the induction of DSBs in chromosomal DNA, endonucleolytic release of Spo11‐oligo complexes frees the DSB ends so that the 5′ strand termini can be exonucleolytically resected to yield extensive 3′ single‐stranded tails that will be coated by the RecA‐related recombinases RAD51 and DMC1 (Figure 3). These nucleofilaments are the key intermediates that mediate the repair of the DSBs by recombination using the sister chromatid or a non‐sister chromatid as template. The invasion of a duplex DNA creates a D‐loop (Petukhova *et al.*, 1998), in which the 3′ end of the invading ssDNA prime DNA synthesis, using the complementary strand as a template (Figure 3).The alternative processing of these joint recombination intermediates will determine its fate as a CO or a NCO product (Szostak *et al.*, 1983). As extensively dissected in yeast and *Arabidopsis* (Mercier *et al.*, 2015) in the major ZMM (*ZIP1, ZIP2, ZIP3, ZIP4, MSH4, MSH5* and *MER3*) pathway, which accounts for 90% of the COs (called Class I COs) in *Arabidopsis*, the extension/displacement of the D‐loop and DNA synthesis can trigger single‐strand annealing on the other side of the DSB, a process called second‐end capture. Further DNA synthesis and ligation will promote the formation of double Holliday junctions (dHJs) that can be resolved into CO recombinant molecules; differently, dHJs can be resolved by dissolution leading to NCOs. An alternative pathway, which accounts for only 10% of the overall COs in *Arabidopsis* (called Class II COs), relies on the resolution of the intermediates by structure‐specific endonucleases that include MUS81 (Berchowitz et al. 2007). This leads to non‐interfering COs. The recombination intermediates can also be resolved as NCOs upon unwinding of the extended invading DNA strand and reannealing to the complementary strand on the second end of the DSB. This mechanism of DSB repair is called SDSA (Strand Displacement Synthesis Annealing) (Pâques and Haber, 1999).

In most eukaryotes, the number of DSBs per meiosis largely exceeds the number of COs (Muyt *et al.*, 2009b). In *Arabidopsis*, for instance, the number of DMC1 foci, diagnostic of the homologous chromosome invasion step that initiates the homology‐directed repair (HDR) in leptotene, reaches 100–200 (Ferdous *et al.*, 2012; Kurzbauer *et al.*, 2012). In contrast, at diakinesis, the number of MLH1 foci (cytological marker of Class I COs) averages only 10, *that is* two per chromosome pair (Muyt *et al.*, 2009b). In maize, at least 500 DSBs are detected as RAD51 foci and their repair yields on average 20 COs per meiotic cell (Sidhu *et al.*, 2015).

Two phenomena regulate the overall number and position of COs: the first one is CO interference, a process that reduces the probability of two COs to occur in vicinity (Berchowitz and Copenhaver, 2010). Notably, the Class I COs are interfering (Drouaud *et al.*, 2013), whereas the Class II COs are not (Henderson, 2012). The second CO regulating phenomena is CO homeostasis, which proportionally enhance the number of COs when the total number of DSBs per meiotic cell is reduced. This homeostatic control appears robust in yeast, worm and mouse (Cole *et al.*, 2012; Hillers and Villeneuve, 2003; Martini *et al.*, 2006). However, it seems more limited in *Arabidopsis* since a significant decrease in COs was observed in *Atspo11‐1* hypomorphic mutants with reduced DSBs (Xue *et al.*, 2018). the CO number per meiosis has been cytologically examined in a panel of maize inbred genotypes. CO number ranged from 11.2 to 19.4 (1.7‐fold variation), maintaining a minimum of one CO per chromosome pair, that is retaining the obligate CO per bivalent. However, beyond this obligate CO, the number of COs was found to correlate with the observed number of DSBs in the panel of genotypes, suggesting that homeostasis may not tightly operate once the obligate COs are ensured (Sidhu *et al.*, 2015).

## Global recombination landscape

The frequency and distribution of COs among and along the chromosomes are uneven, especially in plants (Lambing *et al.*, 2017; Mézard *et al.*, 2015). In *Arabidopsis* and bread wheat, more than 80% of the recombination events occur in 25% of the genome (Choi *et al.*, 2013; Darrier *et al.*, 2017). A consistent higher CO frequency in sub‐telomeric regions has been observed in genomes representing a range of sizes such as those of rice, tomato, maize, barley and bread wheat (Choulet *et al.*, 2014; Demirci *et al.*, 2017; Gore *et al.*, 2009; Higgins *et al.*, 2014; Wu *et al.*, 2003). These recombination‐prone regions next to telomeres are generally hypomethylated, gene‐ and DNA transposon‐rich. Conversely, recombination is suppressed in centromeric and pericentromeric regions which are generally heterochromatic, have a high long terminal repeat (LTR) retroelement content and are gene‐poor (Henderson, 2012). An extreme example is illustrated by the 1‐Gbp‐long bread wheat chromosome 3B, in which nearly all the COs occur in the most distal 13% of the chromosome (Choulet *et al.*, 2014). Genome annotation and transcriptome analysis revealed that fast‐evolving regions distal to the centromere tend to contain genes regulated in a tissue‐specific manner or in response to environmental cues, such as disease resistance gene clusters, whereas more proximal regions contained constitutively expressed and housekeeping genes (Choulet *et al.*, 2014; Pingault *et al.*, 2015). Furthermore, contrasted CO landscapes can be observed in plant male and female meiosis; indeed, in *Arabidopsis*, the total genetic map is 1.7‐fold longer in male meiosis compared to female meiosis, while no chromosome distal increase in COs is observed in female meiosis (Giraut *et al.*, 2011). In maize, coincident CO landscapes with distal increases are observed in both male and female meiosis (Kianian *et al.*, 2018; Luo *et al.*, 2019) although these latter studies reported or not inflation of the male meiosis map. It illustrates species specificities in sex dimorphic CO landscapes.

In cultivated rice, as in *Arabidopsis*, the COs are more evenly distributed along the chromosomes than in larger and more LTR TE‐rich genomes but meiotic recombination remains suppressed at the centromere core and surrounding regions, representing 11% of the total chromosome length (Chen *et al.*, 2002; Wu *et al.*, 2003; Zhao *et al.*, 2002). Localization of heterochromatin in pachytene chromosomes correlates with region with low recombinogenic activity (Cheng *et al.*, 2001). 4′,6‐diamidino‐2‐phenylindole (DAPI)‐bright regions of compacted chromatin indeed largely occupy the short arms of chromosome 4 (Zhao *et al.*, 2002), 9 (Wu *et al.*, 2003)—where rice rRNA loci reside—and 10 (Rice Chromosome 10 Sequencing Consortium, 2003) as well as the pericentromeric regions, which are all recombination‐suppressed regions (Chen *et al.*, 2002; Zhao *et al.*, 2002). Overall, the average chromosomal recombination rate ranges from 3.9 to 4.2 centimorgans (cM) per Mb and, at the local scale (Kbp), varies from 0 to 50 cM/Mb (Si *et al.*, 2015; Wu *et al.*, 2003). Cold spots and hot spots of recombination occur in intergenic and genic regions, respectively (Wu *et al.*, 2003).

Altogether, the multiple regulatory layers controlling meiotic recombination explain the distortion between the genetic and physical distances observed in all species, including the plants. In the recent years, this has been confirmed upon whole‐genome sequencing and the establishment of higher‐resolution genetic maps in polymorphic species, notably in rice (Si *et al.*, 2015; Spindel *et al.*, 2013; Wu *et al.*, 2003). Thus, plant breeding which relies on the creation of novel allele combinations, notably for genes located in recombination‐cold regions, is hampered. Further, in hybrids, recombination is reduced by the presence of polymorphism and structural heterologies (Ziolkowski and Henderson, 2017). Moreover, in rice, exploitation of hybrids between distant varieties from different genetic groups and introgression of traits of interest in elite cultivars from wild relatives is known to be affected by hybrid sterility, a major form of postzygotic reproductive isolation (Ouyang and Zhang, 2013; Shen *et al.*, 2017). In summary, the natural features of the control of recombination rates and its non‐random distribution may result in the low recovery of useful recombinants and the occurrence of linkage drag.

## Local regulation of DSB and CO formation: towards targeting meiotic recombination

Which mechanisms specify the location and frequency of DSB and CO formation in plants remain to be determined. In yeast, a key regulator of DSB hot spots is the methylation of the histone H3‐K4 residue, under the control of the Compass complex (Acquaviva *et al.*, 2013; Borde *et al.*, 2009; Sommermeyer *et al.*, 2013). Slightly differently, in mice and humans, a key regulator of DSB hot spots is the histone methyl transferase PRDM9 that contains a series of DNA‐binding zinc finger domains targeting H3K4me3 to genome sites containing a GC‐rich degenerate DNA sequence motif, thereby similarly controlling chromatin accessibility and initiation of meiotic recombination (Baudat *et al.*, 2010).

No ortholog of PRDM9 has been identified yeasts and plants (Zhang and Ma, 2012), so mechanistically the control of recombination via H3K4 methylation in plants might be more similar to yeast.

Crossover hot spots in plants, like in yeast but contrasting with fission yeast and mice, might be located principally in gene regulatory regions which are known to exhibit an open chromatin structure (Drouaud *et al.*, 2013; He and Dooner, 2009; Yao *et al.*, 2002; Yelina, *et al.*, 2012). The low nucleosome occupancy in gene promoter and terminator sequences favours DSB formation (Choi *et al.*, 2013) Since in this species, COs are closely associated with DSB, this translates into CO hot spots in these regions (Choi *et al.*, 2013). However, this association does not always hold true: in maize, while DSBs occur in all chromosomal regions, with a majority in repetitive DNA, including centromeres and rRNA loci, COs are mainly contributed by DSBs formed in genic regions (He *et al.*, 2017).

DNA and chromatin features have been found linked to DSB localization in plants. In *Arabidopsis*, DSB hot spots are associated with AT‐rich sequences residing in regions with low nucleosome occupancy. These DSB hot spots are correlated with CO hot spots. Three DNA motifs (A‐rich, CCN and CTT) enriched in CO regions, some of which are found around peaks of nucleosome occupancy and of H3K4me3 marks, have been deduced from the analysis of 737 CO events in Arabidopsis (Shilo *et al.*, 2015). In maize, sequencing of 104 CO sites allowed the identification of an associated 20‐bp‐long, GC‐rich degenerated sequence motif with similarity to the DSB Maize Hotspot Sequence (MHS) (He *et al.*, 2017). This motif exhibited similarity to the CCN motif previously identified in *Arabidopsis* (Shilo *et al.*, 2015). An increasing correlation strength was found between genic DSB hot spots and CO sites (*R* ^2^ = 0.4) and MHS and CO sites (*R* ^2^ = 0.7) (He *et al.*, 2017). The MHS DNA cytosine methylation level is thought to be a regulator of hot spot strength, which could explain the modification of the recombination landscape observed in *Arabidopsis* hypomethylated mutants (Mirouze *et al.*, 2012; Yelina *et al.*, 2012).

In *Arabidopsis* and maize, COs tend to co‐localize with H3K4me3 sites (Choi *et al.*, 2013; Kianian *et al.*, 2018) but CO frequency does not strongly correlate with the H3K4me3 site density (Choi *et al.*, 2018; He *et al.*, 2017). In *Arabidopsis*, a link has been established between recombination and the presence of the histone variant H2A.Z which is frequent in promoter regions and responsible for nucleosome mobility (Choi *et al.*, 2013). A reduced frequency of cytosine methylation at CG and CHG sites in a chromosomal region is positively correlated with occurrence of recombination, but this does not hold true at a more local scale. Global loss of cytosine methylation in *Atmet1* increases DSB and CO formation in euchromatic and centromeric regions but decreases them in pericentromeric regions (Choi *et al.*, 2018; Yelina *et al.*, 2012; Yelina et al., 2015a,2015b). On the other hand, loss of methylation at non‐CG, CHG sites through mutation in the DNA methyltransferase *AtCMT3,* increases DSB and COs in pericentromeric regions (Underwood *et al.*, 2018).

Although rice has often been a pioneer species for cereal genomics research, there are surprisingly no published reports of the analysis of DSB‐associated sequences, which could have been retrieved by sequencing chromatin associated to SPO11 or RAD51. Report of whole‐genome sequencing of a large number of segregating progeny plants allowing the identification of motifs associated with COs is scarce (Si *et al.*, 2015). Recently, however, following a training of machine learning models on the 1287 CO positions retrieved from a F2 population of rice (Si *et al.*, 2015), the presence of specific DNA shape structure and low CA dinucleotide frequency was found a predictor of CO occurrence specific to rice (Demirci *et al.*, 2018). Other features favouring COs and shared with other plant species (maize, tomato and *Arabidopsis*) encompass DNA helix twist, and AT, TA, AA and TT dinucleotide frequencies.

While the mechanisms controlling the frequency and the spatial distribution of DSBs in plants remain to be better understood, it may be possible to guide the initiation of recombination to a desired region by targeting the SPO11 protein complex as performed in yeast (Székvölgyi *et al.*, 2015). Historically, fusion of the SPO11 coding region to a GAL4 binding domain (BD) was sufficient to induce DSBs and recombination at GAL4 binding sites, the upstream activating sequences (UAS: CGGN11CCG, where N can be A, T, G or C), in naturally cold regions of the yeast genome (Peciña *et al.*, 2002; Robine *et al.*, 2007). Inactivation of endogenous SPO11 was not necessary to observe the effect, but enhanced stimulation was observed in the *spo11∆* background. A transposition of this strategy is being investigated in rice via the introduction of a T‐DNA carrying a GAL4BD::OsSPO11‐1 fusion driven by the constitutive maize ubiquitin promoter (ZmUbi1) in a distant hybrid genetic background. The number of putative unique GAL4BD recognition sites in the rice genome is greater than 60,000 (vs. 800 putative UAS sites in the yeast genome). Therefore, it is likely that this strategy needs to be implemented in an *Osspo11‐1* mutant background to observe a stimulation of recombination in UAS‐rich regions through the targeting of the SPO11‐1 fusion protein and a possible redistribution of crossovers in the genome (Petit J, Meunier AC, Fayos I, Frouin J, Droc G, unpublished).

Other methods to target the SPO11 protein to the desired site also worked in yeast. Namely, SPO11 was fused to other DNA‐binding domains such as other natural transcription factors, arrays of Zinc finger elements or transcription activator‐like elements (TALEs), or to a catalytically inactive Cas (dead Cas9 (dCas9) (Sarno *et al.*, 2017). A 2.5‐ to 6.3‐fold increase in CO formation was observed near target sites, with the degree of stimulation varying according to the construct and the targeted region. Again, all the fusions were found to retain their ability to form DSBs at natural sites in the *spo11∆* context, so the inactivation of SPO11 is not compulsory to obtain a stimulatory effect (Sarno *et al.*, 2017). The possibility of programming COs at determined, unique sites of a plant genome by the use of a dCas9::OsSPO11‐1 fusion guided by sgRNAs targeting specific regions is being investigated in rice (Fayos I, Meunier AC, Vernet A, Frouin J, unpublished). Once implemented in plants, this technology will be useful to produce a large number of recombinants for map‐based cloning or breaking linkage drag between antagonist genes located in repulsion. As an example of application, the breeding of semidwarf (sd) green revolution cultivars unwittingly dragged a drought‐susceptibility allele in the 500 Kb qDTY1.1 quantitative trait locus (QTL) region together with the favourable allele at the *sd1* locus (Vikram *et al.*, 2015). As a 100 Kb distance on chromosome 1 separates *sd1* from the QTL region targeting recombination in this interval will allow the dissociation of this undesirable linkage.

## Enhancing meiotic recombination

The tight regulation of the overall CO number per meiosis mentioned above prompted research efforts to decipher the molecular control of CO and NCO formation, notably in the model plant *Arabidopsis.* Ethyl methanesulfonate (EMS) mutant screens for restoration of fertility in a ZMM‐deficient genetic background allowed the identification and characterization of several genes that limit COs in these pathways (De Muyt *et al.*, 2009b). The first identified genes were the DNA helicases *FANCM* and *RECQ4a/b* (Crismani *et al.*, 2012; Séguéla‐Arnaud *et al.*, 2015). They function to unwind the post‐invasion recombination intermediates that result in the formation of NCOs through the synthesis‐dependent strand annealing (SDSA) mode of repair. These factors and the associated proteins TOP3⍺ and RMI1 participate in the FANCM and RECQ4a/b pathways, respectively (Crismani *et al.*, 2012; Séguéla‐Arnaud *et al.*, 2015) (Figure 3).

As *FANCM* and *RECQ4* are associated with the Class II CO pathway (not interference‐sensitive), the impact of these mutations and their combination on the CO rate have been extensively investigated in *Arabidopsis* (Crismani *et al.*, 2012; Séguéla‐Arnaud *et al.*, 2015). Mutation of *FANCM* resulted in a nearly threefold increase in recombination frequency in an inbred context, but this effect vanished in a hybrid context (Fernandes, Séguéla‐Arnaud, *et al.*, 2018b). Mutations of *RECQ4a* and *RECQ4b* led to a 5.9‐fold increase in CO frequency, maintained in a hybrid context. The frequency of COs in the double *recq4a/recq4b* and triple *fancm/recq4a/b* mutants was similar in a hybrid context but cumulative (8.8‐fold increase) in a pure line context (Fernandes, Séguéla‐Arnaud, *et al.*, 2018b).

**Figure 4:**
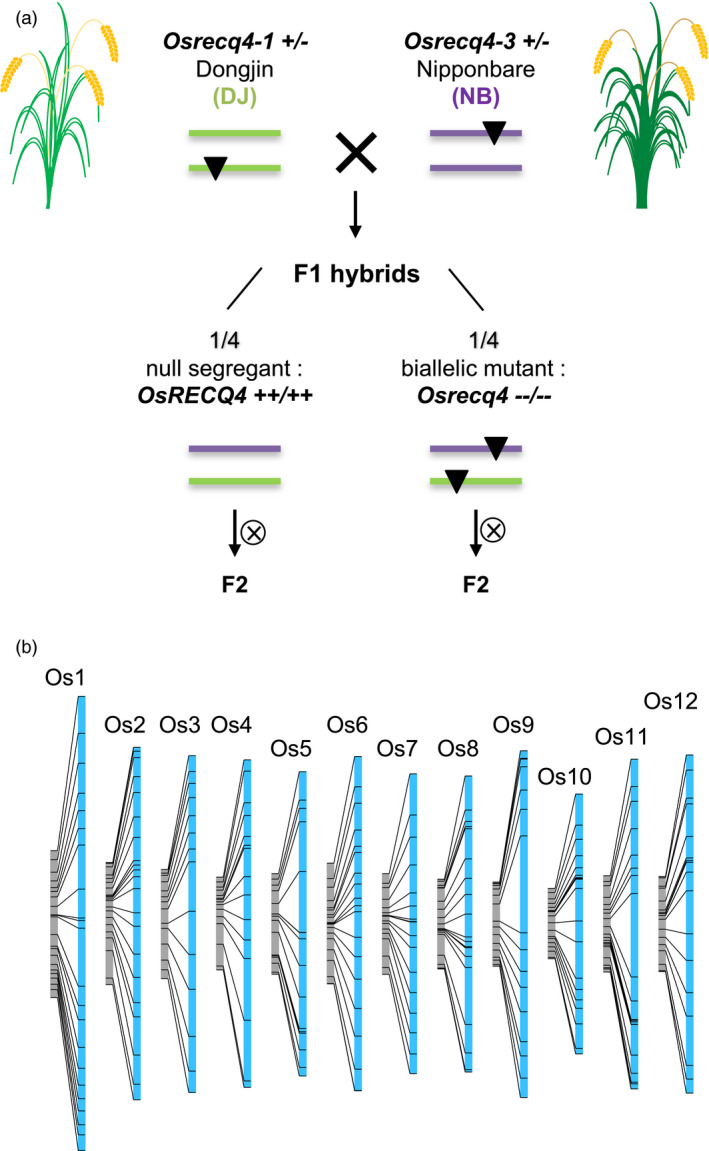
A large increase in recombination is associated with the *recq4* mutation in rice. (a) Generation of a *recq4* biallelic homozygous mutant and its azygous counterpart in the Dongjin (DJ)/Nipponbare (NB) F1 hybrid context. (b) Comparative linkage maps constructed by genotyping 90 loci of F2 populations derived from homozygous biallelic mutant F1s and wild‐type F1s. An average 3.2‐fold inflation of the genetic map is observed in the *recq4* context (Mieulet et al., 2018).

A third gene identified in the same screen, the AAA‐ATPase FIDGETIN‐like 1 (FIGL1), was found to control the dynamics of RAD51 and DMC1 in counteracting interhomolog strand invasion, thereby regulating CO formation early and negatively (Girard *et al.*, 2015). The *figl1* mutation modestly enhances CO frequency in both pure line (1.25×) and hybrid (1.8×) contexts (Girard *et al.*, 2015). However, the addition of a *figl1* mutation in a *recq4a/b* double mutant context significantly increased the genome‐wide CO frequency from a 4.4 fold level to 7.8 fold 4.4‐fold to 7.8‐fold (Fernandes, Séguéla‐Arnaud, *et al.*, 2018b). Remarkably, the triple mutant exhibits 60.7 ± 2.3 COs per meiosis, compared to 7.8 ± 0.4 COs in the wild type (Fernandes, Séguéla‐Arnaud, *et al.*, 2018b). These results prompted to investigate their extension in crops. Similar to most lineages, rice has a single *OsRECQ4* gene, while the duplication observed in *Arabidopsis* appears to be specific to the Brassicaceae and a few other lineages. Two T‐DNA insertion lines carrying allelic mutations of *OsRECQ4*, residing in a Dongjin and Nipponbare temperate *japonica* background, were crossed at a heterozygous state to obtain ¼ null segregant and ¼ biallelic F1 plants (Figure 4). *Osrecq4* F1s exhibited normal fertility and meiosis progression. Genome‐wide genotyping of the two F2 populations at the same diagnostic single nucleotide polymorphism (SNP) markers yielded two genetic maps that could be compared. The *Recq4* mutation increased the recombination frequency 3.2‐fold and the genetic map length from 1759 to 5700 cM. Similar strategies allowed the identification of allelic T‐DNA insertions in the Dongjin and Nipponbare temperate *japonica* backgrounds at the *OsFANCM* locus. The genetic map was enhanced 2.3‐fold in the *Osfancm* mutant (Mieulet *et al.*, 2018). Similar to the pattern observed in *Arabidopsis*, both *Osfancm*‐ and *Osrecq4*‐mediated recombination enhancements were high in regions distal to the centromere but did not operate in centromeric regions, indicating that other mechanisms limiting COs remain to be discovered. Similar stimulating effects of *fancm* and *recq4* mutations were observed in turnip mustard and rapeseed (Blary *et al.*, 2018) and tomato and pea (Mieulet *et al.*, 2018), respectively. Therefore, this approach might be applicable to a large range of crop plants.

In contrast to *Arabidopsis*, the stimulation of *fancm*‐mediated recombination was observed in a hybrid rice context, with the restriction that Dongjin and Nipponbare are two temperate *japonica* cultivars exhibiting a rather low level of interparental polymorphism (estimated to be 1 SNP/11 kb) compared to the *Columbia* and *Landsberg erecta* accessions of *Arabidopsis* (1 SNP/200 bp) (Fernandes, Séguéla‐Arnaud, *et al.*, 2018b; Mieulet *et al.*, 2018). Therefore, investigating the individual and cumulated effects of *Osfancm* and *Osrecq4* mutations in a range of hybrids from closely to distantly related parents will be important. Since the genetic backgrounds in the existing rice mutant libraries are limited, this influence could be tested by generating biallelic mutations at these loci through the introduction of CRISPR‐Cas9 T‐DNA constructs in regenerable tissues of F1 hybrids. Analysis of the stimulation at a more regional scale, notably in chromosomal regions exhibiting increasing genetic polymorphism, presence–absence variation (PAV) or other structural heterologies, or comparison of their epigenetic status (DNA methylation, histone modifications, etc.) also remains to be performed. To note, a representative panel of maize inbred genotypes has indeed been found to exhibit large variation in both DSB (218–608, established from RAD51 foci) and CO numbers (from 11.2 to 19.4, established from observed chiasmata) (Sidhu *et al.*, 2015); although the existence of such variation remains to be established in rice lines and hybrids, the incidence of mutation of anti‐CO genes in a range of genotypes exhibiting such a spectrum of COs must be investigated.

As FANCM and RECQ4 have an established function in DNA stability in somatic tissues and, notably, in meristem maintenance (Kwon *et al.*, 2013), the short‐ and long‐term influence of these mutations on the phenotype should be precisely examined. If a detrimental effect on the phenotype is observed, these mutations should not persist in breeding materials and will need be segregated out. An alternative would be to transiently suppress anti‐CO genes using meiosis‐specific expression of a CRISPR interference T‐DNA construct targeting promoters of anti‐CO genes in F1 meiocytes, which can be segregated out in later generations of breeding.

As said, since the association of *figl1* and *recq4a/b* provided the highest stimulatory effect on genome‐wide recombination in *Arabidopsis*, it was tempting to explore the same combination of mutations in rice. However, a report indicated that the rice *figl1* mutant exhibits a complete sterility phenotype that precludes its further utilization for recombination enhancement (Zhang *et al.*, 2017). A similar result was observed in pea (Mieulet *et al.*, 2018). These observations indicating species‐to‐species differences in mutant viability and downstream effects on fertility temper the direct extension of the *Arabidopsis* results in every plant but a solution might be to identify hypomorphic mutants. Similarly, partial inactivation of the transverse filament protein of the synaptonemal complex, ZEP1 (Wang *et al.*, 2010), which is orthologous to *Arabidopsis* ZYP1, allowed a 1.8‐fold increase in Class I CO frequency but partially altered fertility in rice (Wang *et al.*, 2015).

Alternative cumulating strategies could be used. For instance, ectopic expression of the pro‐crossover E3 ligase protein of the ZMM pathway HEI10 (Ziolkowski *et al.*, 2017) was recently found to act additively with *recq4a/b* to enhance the frequency of recombination in *Arabidopsis* from 7.5 to 31 COs per F2 individual (Serra *et al.*, 2018). As the HEI10 ortholog has been identified and characterized in rice (Wang *et al.*, 2012), extension into rice can be attempted.

A remaining challenge is to enhance CO frequency in regions close to centromeres in order to gain access to the underlying genes of interest. A promising avenue is to alter repressive epigenetic marks, including DNA methylation, of H3K9me2, H3K27me1 and H2A.W, which may have differentiated roles in the control of recombination in these regions. Epigenetic activation of meiotic DSBs in proximity to centromeres occurs in a DNA Methyl Transferase 1 (MET1) *Arabidopsis* homozygous mutant (Choi *et al.*, 2018) and parallels reduced nucleosome occupancy, a gain of transcription and the occurrence of recombination‐favourable H3K4me3 marks in the stimulated regions. Extensive remodelling of CO frequency with elevated COs in regions proximal to the centromere coinciding with pericentromeric decreases and distal increases is observed in hypomethylated plants, while the total number of COs remains unaltered (Yelina *et al.*, 2012). In *Arabidopsis*, mutation of the H3K9 methyltransferase genes KYP/SUVH4‐5‐6 or the CHG DNA methyltransferase CMT3 increases meiotic recombination in proximity to centromeres and pericentromeres in both inbred and hybrid contexts, likely involving the contribution of Class I and Class II CO repair pathways (Underwood *et al.*, 2018). Conversely, it is possible to impose, via the RNA‐directed DNA methylation (RdDM) pathway, DNA methylation of endogenous *Arabidopsis* meiotic CO hot spots located in euchromatin, which is associated with the gain of H3K9me2 and increased nucleosome occupancy (Yelina et al., 2015a,2015b).

## Abolishing meiotic recombination to engineer apomixis

Whereas most plant species reproduce by seeds through meiosis and double fertilization, species of more than 120 angiosperm genera reproduce asexually by seeds in a clonal manner, a mechanism called apomixis. The most achieved apomictic pathway is gametophytic diplospory, which bypasses meiosis and triggers parthenogenetic development from an unreduced megaspore, thereby producing embryos harbouring the full maternal chromosome complement (Hand and Koltunow, 2014). Although this mode of reproduction has its own advantages, it also abolishes the genetic variation resulting from recombination, which is a major source of diversity and adaptation. Nevertheless, most natural apomictic plant species are actually facultative and can accomplish normal sexual reproduction (Hand and Koltunow, 2014).

A highly desirable goal in crops is to obtain clonal reproduction through seeds as it will allow the release of immortalized hybrids with fixed hybrid vigour (heterosis) (Khush *et al.*, 1998). However, despite an initial belief in the simple genetic structure of apomixis, the transfer of this mode of reproduction to crops remains unachieved (Ronceret and Vielle‐Calzada, 2015). Due to an autogamous mode of reproduction, rice varieties are mainly pure lines, but the existence of 20% yield heterosis has prompted the development of F1 hybrids, which represent 20% of the global rice acreage and up to 60% of the rice acreage in China (Cheng *et al.*, 2007). The generation of F1 seeds relies on thermosensitive and photoperiod‐sensitive male sterility or genocytoplasmic male sterility and complex, labour‐intensive hybridization schemes (Fan and Zhang, 2018; Tang *et al.*, 2017). For these reasons, hybrid seeds have an extra cost that subsistence farmers cannot afford. Engineering apomixis in rice would allow the immortalization of F1 hybrids that could clonally propagate via seeds. However, despite intensive efforts in the 1980s, no source of apomixis has been discovered in either rice or its wild relatives (Khush *et al.*, 1998).

The various forms of apomixis include 3 common developmental components: (i) a bypass of meiosis during embryo sac formation; (ii) development of an embryo in a fertilization‐independent manner; and (iii) formation of viable endosperm in a fertilization‐dependent or fertilization‐independent manner (Hand and Koltunow, 2014). In the gametophytic category of apomixis, the embryo sac is formed through mitosis from a diploid cell of the ovule (apomeiosis), thereby bypassing meiosis, and the diploid cell develops into an embryo without fertilization. There are two types of apomeiotic development based on the origin of the diploid cell that gives rise to the embryo sac: diplospory and apospory. In diplosporous apomeiosis, the precursor is the megaspore mother cell (MMC) (the mode found, for instance, in *Tripsacum*), whereas in aposporous apomeiosis, the precursor is a diploid somatic cell adjacent to the megaspore mother cell (the mode found, for instance, in *Pennisetum*). Formation of the endosperm may necessitate the fertilization of the central cells of the embryo sac by a sperm nucleus (Hand and Koltunow, 2014).

In diplosporous apomeiosis, which appears to be the most desirable first component for engineering synthetic apomixis in crops, megaspore meiosis is turned into mitosis. There are 3 essential differences between meiosis and mitosis: (i) induction of chromosomal DSBs in prophase of meiosis I that result in homologous chromosome pairing and recombination; (ii) migration of the homologous chromosomes to opposite poles in meiosis I; and (iii) occurrence of a second division that separates sister chromatids. Several meiotic mutants that develop an embryo sac without meiosis II have been characterized in plants. The maize mutant *elongate 1* (*el1*) controls a single equational division and the arrest of further progression after meiosis I, forming unreduced but recombined diploid gametes, with one dyad directly initiating embryo sac development (Barrell and Grossniklaus, 2005). However, *el1* actually forms both diploid and haploid functional embryo sacs. In maize, the production of viable unreduced gametes without meiosis in a diplospory‐like developmental pathway has been observed in a mutant of the *AGO104* gene. AGO104 modifies chromatin through DNA methylation that would repress somatic fate in germ cells (Singh *et al.*, 2011). The *Arabidopsis dyad* mutant allele of SWITCH1, a nuclear coiled protein essential for meiotic entry, leads to the formation of functional apomeiotic female gametes at a low frequency but considerably reduces female fertility (Ravi *et al.*, 2008) and is therefore not amenable to building synthetic apomictic crops.

An elegant alternative strategy implemented in *Arabidopsis* targets each of the three key elements discriminating meiosis from mitosis via the construction of the *Mitosis instead of Meiosis* (*MiMe*) mutant genotype (d’Erfurth *et al.*, 2009). *MiMe* is produced through a combination of mutations in three meiosis‐specific genes. First, inactivation of the topoisomerase‐like AtSPO11‐1 transesterase prevents programmed DSB formation and abolishes subsequent homologous chromosome pairing and meiotic recombination (Grelon *et al.*, 2001). Second, the mutation of the cohesin REC8 causes the separation of the sister chromatids during the first meiotic division (Chelysheva *et al.*, 2005). Lastly, mutation in omission of second division 1 (OSD1), a plant‐specific protein promoting meiotic progression through anaphase‐promoting complex/cyclosome (APC/C) inhibition, causes the second meiotic division to be skipped (Cromer *et al.*, 2012). Meiosis in *MiMe* occurs without recombination and distributes sister chromatids during a single division, mimicking the process in mitosis. *MiMe* therefore produces clonal male and female diploid gametes, doubling the chromosome complement in each selfing generation (up to octoploid progeny). It was subsequently shown that alternative mutations in other genes essential for the initiation of recombination through DSB formation (e.g. *PRD1*, *PRD2* or *PRD3/PAIR1*) can be used instead of *SPO11‐1* in combination with *REC8* and *OSD1* to generate the *MiMe* phenotype (Mieulet *et al.*, 2016). Similarly, mutation of TARDY ASYNCHRONOUS MEIOSIS (TAM), an A‐type cyclin (CYCA1;2) essential for preventing meiosis termination at the end of the first division, as well as a dominant mutation in THREE DIVISION MUTANT 1 (TDM1), also leads to a premature exit from meiosis after the first division and can be used instead of *osd1* (Cifuentes *et al.*, 2016; d’Erfurth *et al.*, 2010).

The *MiMe* genotype has been reproduced in rice by crossing insertion lines harbouring heterozygous mutations in either *PAIR1*, *OsREC8* or *OsOSD1* (Mieulet *et al.*, 2016). At the initiation of the work, while the meiotic functions of PAIR1 (Nonomura *et al.*, 2004) and OsREC8 (Shao *et al.*, 2011) were clearly established, there was a need to discriminate OsOSD1 from a putative paralog. *Arabidopsis* indeed harbours an OSD1 paralog, UVI4, which has a distinct function in regulating the somatic cell cycle. In rice, both groups of genes exist, making distinction by sequence homology difficult, but a single mutation in *Os02g37850* was found to be sufficient for observing the meiotic defects of *Arabidopsis osd1*. A single gene orthologous to *OsOSD1* was readily identified in the barley and *Brachypodium* genomes, whereas the maize, sorghum and *Setaria* (Andropogoneae) genomes harboured a tandem duplication of *OSD1* (Lloyd *et al.*, 2014). This finding indicates that the *MiMe* phenotype can be generated in other cereals.

**Figure 5:**
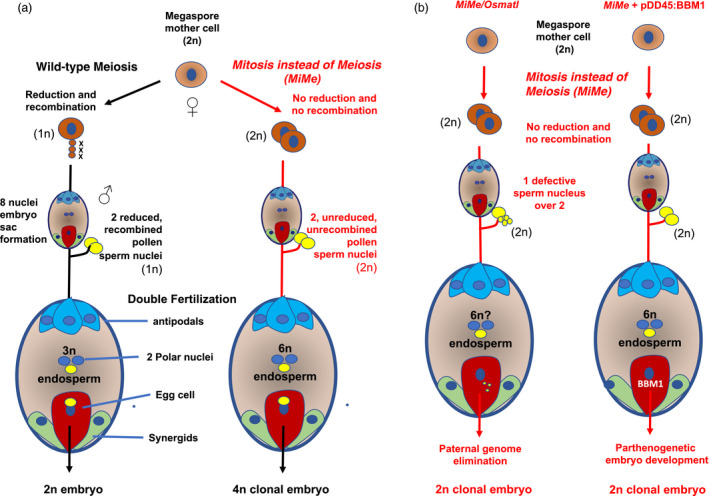
Sexual reproduction and synthetic apomixis in rice. (a) In the triple homozygous mutant *pair1/rec8/osd1* (*MiMe*), the Megaspore Mother Cell (MMC) undergoes a mitosis instead of a meiosis generally leading to the formation of unreduced and unrecombined diploid (2n) male and female gametes. A tetraploid (4n) clonal embryo is formed through the fusion of the 2n egg cell nucleus and a 2n pollen sperm cell nucleus and is generally surrounded by a hexaploid (6n) endosperm. The resulting seeds are viable and form 4n clonal plants (Mieulet et al., 2016). (b) Using *MiMe* as a common background, two strategies have been implemented to achieve synthetic apomixis: (i) in the first strategy, *Osmatl* is combined with *MiMe*. A homozygous mutation in the *OsMATL* gene, orthologous to the maize *MATRILINEAL/NLD/ZmPLA1* gene, provokes chromosome elimination in one or both sperm nuclei participating in the double fertilization. This conducts to the formation of ca. 6% haploid embryos in self‐pollinated *Osmatl* rice plants (Yao et al., 2018). In the *MiMe/Osmatl* quadruple mutant, a 2n clonal embryo is formed, representing 5%–9% of the viable seeds (Wang et al., 2019). (ii) In the second strategy, transgenic accumulation of rice BABY BOOM1 in an egg cell‐specific manner and in the *MiMe* context triggers a parthenogenetic development of the 2n egg cell into a clonal embryo with a 9%–29% frequency (Khanday et al., 2019).

In the rice *MiMe* triple mutant, meiosis was converted into a mitotic‐like division with balanced segregation of sister chromatids in a single division. Similar to *Arabidopsis*, rice *MiMe* plants produced diploid male and female gametes genetically identical to their parent at an estimated 100% and 85% frequency, respectively, with no major impact on fertility. MiMe progeny seeds harbour both unreduced and unrecombined chromosome complement (Figure 5a). Diploid *MiMe* egg cell is therefore an ideal material for introducing parthenogenesis, the second component of apomixis, which is the initiation of embryo formation from an unreduced egg cell without its fertilization. However, no connection has been established between unreduced gamete formation and the triggering of parthenogenetic development from the egg cell (Ronceret and Vielle‐Calzada, 2015). One possible method for triggering parthenogenesis is to cross with a line in which the genome is eliminated following fertilization. The centromere‐specific histone CENH3 null mutant coexpressing altered CENH3‐GFP variant constructs, called *GEM* (for Genome Elimination induced by a Mix of CENH3 variants), was used first (Ravi and Chan, 2010). In *Arabidopsis*, crossing either *dyad* or *MiMe* as a female parent with a *GEM* line allowed the generation of clonal diploid plants at a 13% and 34% frequency, respectively (Marimuthu *et al.*, 2011). This report represents the first breakthrough in synthetic apomixis. However, the number of viable seeds per fruit remained low, notably in *dyad* (0.9 seeds per pod) (14 seeds per pod in *MiMe*), and the system still necessitates crossing and cannot be autonomously reproduced by self‐propagation.

Recently, the causative gene responsible for the ability of some maize lines to trigger haploid induction by gynogenesis at an 8%–10% frequency was independently isolated and characterized by three research teams (Gilles *et al.*, 2017; Kelliher *et al.*, 2017; Liu *et al.*, 2017). The gene, a patatin‐like phospholipase A called NOT LIKE DAD (*NLD*), MATRILINEAL or *ZmPLA1,* has activity restricted to the pollen tube, and the inducer allele contains a frameshift mutation, the phenotype of which can be reproduced by CRISPR‐Cas9 (Gilles *et al.*, 2017; Kelliher *et al.*, 2017). It has been shown that the mutation is associated with sperm chromosome fragmentation, which may result in, among other events, paternal genome elimination in the fertilized egg cell (Li *et al.*, 2017).

The rice ortholog of NLD, *OsMATL,* has been characterized and its mutation allowed the development of haploid‐induced lines in *indica* rice (Yao *et al.*, 2018). This finding is of great interest because *indica* rice varieties have poor amenability to doubled‐haploid generation through anther and pollen culture (Silva, 2010). Furthermore, cumulating *MiMe* and *OsMATL* mutations by simultaneously targeting the four genes should be possible since the rice genome is highly amenable to both multiplexed Cas9 and Cpf1 mutagenesis, with biallelic lesions induced at a high frequency in primary transformants (Lowder *et al.*, 2015; Wang *et al.*, 2017) (Figure 5b). For a proof of concept of transmission of heterozygosity and heterosis through synthetic apomixis, an experiment would have to be implemented in an F1 hybrid context. This strategy has been recently exemplified in an elite intersubspecific hybrid rice line called Chunyou84 (CY84) (Wang *et al.*, 2019). This achievement is an obvious breakthrough. The panicle fertility of the quadruple mutant was, however, reduced to 4.5% by the *Osmatl* mutation. In *Osmatl*, chromosome elimination in the sperm nucleus which fuse with the central nuclei may indeed affect endosperm development in part of the fertilization events. Among the 145 collected seeds, 9 proved to be diploid, heterozygous and apomictic, while the remaining seeds were tetraploids and nonapomictic. This *MiMe/matl* quadruple mutation strategy has been also been recently implemented using *Osspo11‐1* rather than *pair1* for abolishing homologous chromosome pairing and recombination, thereby reproducing the original *Arabidopsis MiMe* mutant composition (Xie *et al.*, 2019). Unless enhanced phenotypic penetrance from the current 5%–6% haploid seed induction frequency is obtained by further manipulation of *OsMATL*, it is unlikely that this strategy will allow the 100% apomictic seed production that is needed for clonal reproduction of F1 hybrids.

An alternative strategy is therefore to engineer parthenogenesis in *MiMe* (Figure 5b). Candidates include the genes able to induce embryogenesis from somatic tissues that have been identified in *Arabidopsis* in the last 15 years. These include transcription factors involved in cell differentiation, notably the *AP2*/*ERF* gene BABYBOOM (*BBM*) (Boutilier *et al.*, 2002; Horstman *et al.*, 2017), WUSCHEL (Zuo *et al.*, 2002), LEAFY COTYLEDON 1 and 2 (Lotan *et al.*, 1998; Stone *et al.*, 2001) and RKD4 (Waki *et al.*, 2011) or some LRR‐RLK receptors such as the *SERK* genes (Hecht *et al.*, 2001). Transient expression of *BBM* and *WUS2* in cereal calluses has allowed the enhancement of regeneration and transformation ability in cereals and the widening of the genetic pool amenable to transformation (Lowe *et al.*, 2016). Interestingly, a strong candidate gene residing in the heterochromatic, transposable element‐rich and telomeric apospory‐specific genomic region (ASGR) delineated by genetic mapping in the *Pennisetum* apomictic species contains a gene with high sequence similarity to the rice BBM (Conner *et al.*, 2015). Introduction of PsASGR‐BBML constructs in rice and maize promoted parthenogenesis and produced haploid progenies, albeit at variable and generally low frequencies. Plants harbouring a PsASGR‐BBML promoter‐GUS exhibited GUS activity in the ovaries, egg apparatus of the sexual embryo sac and developing embryos (Conner *et al.*, 2017). An attractive strategy to efficiently trigger synthetic apomixis is to engineer *MiMe* with the rice *BBM* driven by more specific egg cell promoters. A combination of expression of such a parthenogenesis‐inducing cassette with a CRISPR‐Cas9 construct simultaneously targeting the 3 *MiMe* components (*pair1, Ososd1* and *Osrec8*) has recently been achieved in rice (Khanday *et al.*, 2019) (Figure 5b). Ectopic expression of *BBM1* driven by the *Arabidopsis* DD45 egg cell‐specific promoter led to the production of up to 29% haploid seeds in nonemasculated flowers of the rice cultivar Kitaake. The three *MiMe* component genes, namely *PAIR1, OsREC8* and *OsOSD1*, were then inactivated through CRISPR‐Cas9 in haploid and diploid seed embryo‐derived calluses harbouring the *pDD45::BBM1* parthenogenetic trigger construct. The cultured cells exhibiting homozygous biallelic editions at the 3 loci regenerated plants that, respectively, formed haploid and diploid apomictic progenies for several generations. The frequency of formation of apomictic seeds was partial but stable across generations, ranging from 10% to 30%, depending on the event. In diploid apomictic seeds, the endosperm is 6n, while the embryo is 2n, a 3:1 ploidy ratio differing from that found in wild‐type seeds (3:2) but sufficient to ensure normal seed formation. Enhancing the current frequency of apomictic seed formation in the *MiMe* mutant could be achieved by refining *BBM1* expression in egg cells and/or by combining the action of several factors that could further increase the efficiency of the parthenogenetic trigger. Recently, the homeobox gene *BELL1*, a master regulator of the gametophyte‐to‐sporophyte transition, was identified in the moss *Physcomitrella patens,* and it has been shown that its ectopic expression induces embryo formation and diploid sporophytes from specific gametophyte cells without fertilization (Horst *et al.*, 2016). Attempting *PpBELL1* expression in *MiMe* egg cells would be an attractive alternative assay for triggering parthenogenesis and apomixis in rice*.*

## Conclusion and prospects

Recent breakthroughs were made in the manipulation of meiotic recombination in the model plant rice. Together with a deeper understanding of the function of meiosis genes (Luo *et al.*, 2014) and of the role of epigenetic factors in the control of recombination (Yelina et al., 2015a,2015b), these progresses pave the way to demonstrate that although naturally very limited in plants as in other eukaryotes, meiotic recombination is somehow flexible. Along this line, improving the existing approaches and expanding our toolbox is a reasonable and likely successful experimental strategy.

Mutations of anti‐CO genes are a powerful tool to increase the CO frequency in rice. The combination of mutations in anti‐CO genes and examination of the local influence of parental genomic polymorphisms and structural variations on recombination enhancement remain to be investigated. Additionally, the putative long‐term detrimental effect of these mutations must be precisely examined. Integration of these mutations into breeding populations followed by field evaluation will allow us to determine to what extent recombination enhancement is translated into the capture of new allelic combinations and an extended range of and added value in agronomic trait variation. In distant intergroup or interspecific crosses of rice, the enhancement of meiotic recombination mediated by mutation of anti‐CO genes should be combined with manipulation of genes controlling postzygotic reproductive isolation to achieve their full potential (Ouyang *et al.*, 2010; Ouyang and Zhang, 2013; Shen *et al.*, 2017). Targeting recombination by directing SPO11 to specific regions of the rice genome should also become a reality in the near future.

Engineering synthetic apomixis in rice is becoming increasingly likely, as demonstrated by the recent proofs of concept of generation of an autonomous system for producing synthetic apomictic seeds (Khanday *et al.*, 2019; Wang *et al.*, 2019). These breakthroughs pave the way for further improvements and application to other crops. Implementation of recombination enhancement and targeting as well as synthetic apomixis in actual breeding materials will be made possible and greatly facilitated by the CRISPR‐Cas9 technology that has been applied in rice in the last six years (Mishra *et al.*, 2018). It will be important to evaluate the field performance of and transmission of heterosis in apomictic hybrid rice. Further enhancement of the apomictic seed recovery frequency, notably through a deeper understanding of the genetic and epigenetic factors controlling early zygote development, should be achievable in the near future.

## Conflict of interest

The authors declare no competing interests.

## Author contributions

IF, DM and JP drafted sections of the manuscript and prepared figures. EG wrote the manuscript core and prepared complementary figures. AN, ACM and CP made a critical revision of the content of the manuscript. All authors contributed to the final reading and approved the submitted version.
